# Treatment of unresectable intrahepatic cholangiocarcinoma with yttrium-90 radioembolization: A systematic review and pooled analysis

**DOI:** 10.1016/j.ejso.2014.09.007

**Published:** 2015-01

**Authors:** D.P. Al-Adra, R.S. Gill, S.J. Axford, X. Shi, N. Kneteman, S.-S. Liau

**Affiliations:** a Department of Surgery, University of Alberta, Edmonton, AB, Canada; b St. George's University, University Centre, Grenada, West Indies; c Center for the Advancement of Minimally Invasive Surgery, Royal Alexandra Hospital, Edmonton, AB, Canada; d Hepatopancreatobiliary Surgical Unit, University Department of Surgery, Addenbrooke's Hospital, University of Cambridge, United Kingdom

**Keywords:** Cholangiocarcinoma, Radioembolization, Yttrium-90 microsphere

## Abstract

Radioembolization with yttrium-90 microspheres offers an alternative treatment option for patients with unresectable intrahepatic cholangiocarcinoma (ICC). However, the rarity and heterogeneity of ICC makes it difficult to draw firm conclusions about treatment efficacy. Therefore, the goal of the current study is to systematically review the existing literature surrounding treatment of unresectable ICCs with yttrium-90 microspheres and provide a comprehensive review of the current experience and clinical outcome of this treatment modality. We performed a comprehensive search of electronic databases for ICC treatment and identified 12 studies with relevant data regarding radioembolization therapy with yttrium-90 microspheres. Based on pooled analysis, the overall weighted median survival was 15.5 months. Tumour response based on radiological studies demonstrated a partial response in 28% and stable disease in 54% of patients at three months. Seven patients were able to be downstaged to surgical resection. The complication profile of radioembolization is similar to that of other intra-arterial treatment modalities. Overall survival of patients with ICC after treatment with yttrium-90 microspheres is higher than historical survival rates and shows similar survival to those patients treated with systemic chemotherapy and/or trans-arterial chemoembolization therapy. Therefore, the use of yttrium-90 microspheres should be considered in the list of available treatment options for ICC. However, future randomized trials comparing systemic chemotherapy, TACE and local radiation will be required to identify the optimal treatment modality for unresectable ICC.

## Introduction

Intrahepatic cholangiocarcinoma (ICC) is a malignant transformation of cholangiocytes within the hepatic parenchyma. The incidence of this primary liver malignancy is increasing,^1,2^ and ICC accounts for up to 15% of primary liver cancers.^3^ In contrast to the other two locations of cholangiocarcinoma (hilar and distal bile duct), intrahepatic lesions are often asymptomatic and, therefore, present as an incidental mass lesion without jaundice or other stigmata of biliary obstruction.^4^ Given the asymptomatic nature of many ICCs, patients often present with locally advanced tumours. Although surgery offers the highest curative potential, many tumours are deemed unresectable at the time of diagnosis.^5^ Patients with unresectable ICC have a median survival of less than eight months.^6,7^ Systemic chemotherapy with gemcitabine and cisplatin offers an overall survival advantage in patients with advanced biliary cancer^8^; however, given the aggressive nature of cholangiocarcinoma and overall poor prognosis, other treatment modalities are being investigated.^9–11^

Radioembolization with yttrium-90 microspheres offers an alternative radiotherapy option for primary and secondary intrahepatic tumours.^12^ In this treatment modality, β-emitting yttrium-90 microspheres are injected into the hepatic artery feeding the tumour, become trapped in the tumour and emit local internal radiation. The advantage of radioembolization is the ability to deliver high dose radiation to the tumour with minimal collateral damage to the normal liver parenchyma or surrounding tissues.^13^ In contrast, non-selective external beam radiation has higher rates of radiation-induced liver disease as normal hepatic tissue is radiated in addition to the tumour. Recently, yttrium-90 radioembolization for the treatment of hepatocellular carcinoma showed a longer time-to-progression and fewer side-effects than trans-arterial delivered chemotherapy protocols.^14,15^ In addition, high response rates were seen with radioembolization treatment of intrahepatic neuroendocrine tumours.^16,17^

Patients with unresectable cholangiocarcinoma have limited treatment options with only modest survival advantages.^8,11^ Local therapy with yttrium-90 microspheres offers the promise of delivering a high dose of radiation directly to the tumour, thereby causing increased tumour destruction. Treatment of ICCs with radioembolization has been attempted; however, only small trials have been performed with this novel treatment. In addition, the relative rarity of ICCs and heterogeneity of this disease makes it difficult to draw firm conclusions about treatment efficacy. The goal of the current study is to systematically review the existing literature surrounding treatment of unresectable ICCs with yttrium-90 microspheres with the aim of providing a comprehensive review of the current experience and clinical outcome of this treatment modality.

## Methods

### Inclusion criteria for considering studies for this review

#### Study characteristics

Given the rarity of yttrium-90 radioembolization treatment for unresectable ICC, studies with greater than one patient were included in order to ensure the comprehensive capture of the available clinical experience. This included human case-series (>1 case), randomized controlled trials, non-randomized controlled trials, prospective cohort series.

#### Participants

The target population consists of adult (>18 years old) male or female patients with unresectable ICC.

#### Interventions

The intervention under study is radioembolization therapy with yttrium-90 microspheres. The yttrium-90 microsphere treatment may be performed before, synchronously, or after systemic chemotherapy.

### Outcome measures

#### Primary outcomes

The primary outcomes are overall survival and radiological response to radioembolization therapy with yttrium-90 microspheres.

#### Secondary outcomes

The secondary outcomes of this study are the ability of yttrium-90 treatment to convert unresectable cholangiocarcinoma to resectable, mortality, and morbidity.

### Search methods for identification of studies

#### Electronic searches

Published English-language manuscripts were considered for review with inclusion from 2000 to 2013. A comprehensive search of electronic databases (e.g., MEDLINE, EMBASE, SCOPUS, BIOSIS Previews and the Cochrane Library) using broad search terms was completed. The bibliographies of all included articles were examined to identify additional potentially relevant publications. Search terms included unresectable intrahepatic cholangiocarcinoma, advanced biliary tract cancer, microsphere, SIR-Spheres, Selective Internal Radiation, TheraSphere, yttrium-90, radioembolization and radiation lobectomy.

### Data collection and analysis

#### Selection of studies

All studies involving radioembolization therapy with yttrium-90 microspheres for unresectable ICCs were included. Given the rarity of studies focussing on yttrium-90 microsphere treatment, manuscripts published in abstract form were included. A dedicated search for the full-length manuscripts of published abstracts was also undertaken. A trained librarian conducted the electronic searches (X.S.), and one author (D.A.) conducted a pre-screen to identify the articles clearly irrelevant articles by title, abstract and keywords of publication. Following this, three independent reviewers (R.G., S.A. and D.A.) assessed the studies for relevance, inclusion, and methodological quality. Articles were classified as either:

1 Relevant (meeting all specified inclusion criteria);
2 Possibly relevant (meeting some but not all inclusion criteria);
3 Rejected (not relevant to the review).

Three reviewers (R.G., S.A. and D.A.) independently reviewed full-text versions of all studies classified as relevant or possibly relevant. Disagreements were resolved by re-extraction, when necessary. In the cases of numerous publications by the same institution, personal communication with the corresponding authors of those studies was undertaken to ensure the uniqueness of the patients in each study.

#### Data extraction and management

Two reviewers (D.A. and X.S.) independently extracted data from the full versions of the manuscripts. The extracted information included details of methods (e.g., randomization, blinding, etc.), demographics (e.g., age, sex, etc.), treatment characteristics (e.g. target dose, number of treatments, etc.), clinical characteristics of each group, study inclusion and exclusion criteria, number of patients excluded and lost to follow-up, baseline and post-intervention outcomes (e.g., median survival, etc.), mortality/morbidity data (e.g., death, abdominal pain, length of hospital stay, etc.) and methods of analysis.

#### Statistical analysis

Pooled analysis was performed on the data from included studies. Descriptive statistics (simple counts, means, and medians) were used to report study, patient- and treatment-level data. The number of patients enrolled was used in the calculation of study and patient demographics. Efficacy outcomes of interest were synthesized by pooling data for patients that underwent therapy involving radioembolization with yttrium-90 microspheres. Due to the high heterogeneity among the studies and lack of randomized controlled trials, a meta-analysis was not deemed appropriate. Statistical calculations were performed using Stata 10 (StataCorp LP, USA) and weighted overall survival and response analysis were performed using Prism 5 (GraphPad Software, San Diego, CA).

## Results

### Results of the search

**Figure 1:**
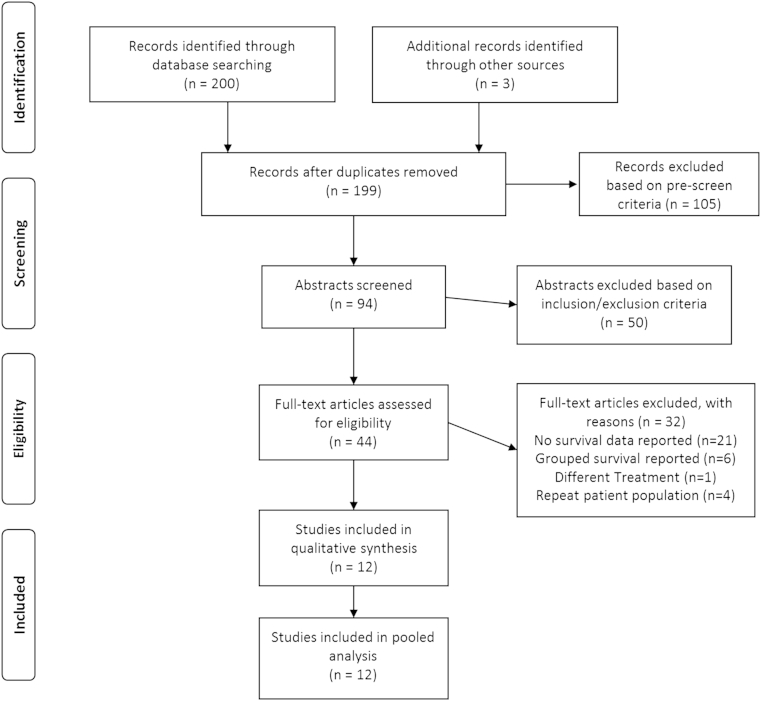
PRISMA flow diagram showing selection of articles for review.

A total of 203 articles were identified using our search criteria for screening (Fig. 1). Following assessment by our exclusion criteria, 105 were rejected and 94 studies remained for abstract review. Following abstract review, 50 studies were excluded and 44 studies remained for full-text eligibility assessment. 12 primary studies meeting the inclusion criteria were identified following thorough assessment of the complete manuscripts^18–29^ (Table 1). These included seven prospective case series^19,20,22,24–26,28^ and five retrospective cohort studies.^18,21,23,27,29^

### Included studies

11 of the 12 included studies contained median survival data for patients undergoing radioembolization therapy with yttrium-90 microspheres for the treatment of unresectable ICC. Outcome data on mortality, morbidity and complications were available from eight included studies. Baseline characteristics of patients in the included studies are provided in Table 1. A total of 298 patients were assessed in the 12 studies and numbers of patients in each study ranged between 2 and 46. The weighted mean age of the patients was 62.1 years, ranging from 57 to 68. The patients had a median follow-up of 10.8 months (range: 6–29 months).

### Characteristics of patients and radioembolization therapy with yttrium-90 microspheres

Details of radioembolization therapy with yttrium-90 microspheres are provided in Table 2. The majority of patients included in the analysis had previously undergone some form of treatment for their ICC prior to radioembolization. Most of these patients previously received chemotherapy (54%) and/or underwent surgical resection (33%). Once patients were deemed refractory to chemotherapy or had unresectable recurrences after initial surgical resection, they were then considered for radioembolization therapy. It was unclear from most studies if chemotherapy was given during or after radioembolization therapy. There was no preference to the type of yttrium-90 delivery system (glass or resin) used to treat the ICC. Based on four included studies, the weighted mean number of treatments per patient was 1.5 with a weighted mean dose of 1.6 GBq.

### Primary and secondary outcomes

The primary outcome, survival, was assessed as median survival. Overall weighted median survival was 15.5 months (range: 7–22.2), based on 11 included studies (Table 3). One study,^27^ did not statistically reach a median survival because data censoring did not allow the survival curve to go below 0.55. However, the mean survival of 17.7 months was close to where the median survival would be should another event occur. Therefore, the nine patients in this study were included in the pooled analysis.

Most commonly, the response evaluation criteria in solid tumours (RECIST) was reported. RECIST is defined as complete response (disappearance of all target lesions), partial response (decrease ≥30% in the sum in the greatest dimension of target lesions), stable disease (decease <30% or increase <20% in sum in greatest dimension of target lesions) and progressive disease (increase ≥20% in sum in greatest dimension of target lesions and/or progression of non-target lesion).^28^ Due to the small number of studies that reported RECIST, these studies were pooled with those reporting the modified (m)RECIST and Positron emission tomography response evaluation criteria in solid tumour (PERCIST; Table 3). Once these studies were pooled, radiological response for solid tumours was reported in six studies. A weighted mean partial response was seen in 28% and stable disease was seen in 54% of patients at three months.

Secondary outcomes included the ability to convert unresectable to resectable disease, mortality, overall morbidity and type of morbidity (Tables 3 and 4). The ability to offer surgical resection to previously unresectable disease was reported in three studies.^24,25,28^ Combined, these studies had a total 73 patients, and surgery was performed on seven patients post-radioembolization. Mortality data was specifically reported in three included studies, and of these, there was one treatment-related death. Overall morbidity was reported in eight included studies and is summarized in Table 4. The most common types of morbidity following radioembolization therapy with yttrium-90 microspheres were fatigue (33%), abdominal pain (28%) and nausea (25%).

## Discussion

Locally delivered radiation with yttrium-90 microspheres is a novel therapy for patients with unresectable ICC who, otherwise, have limited treatment options. The published experience with radioembolization for the treatment of cholangiocarcinoma is narrow, and of these studies, few document survival outcomes. Furthermore, there are no trials directly comparing the efficacy of yttrium-90 microspheres to other available treatment options, such as systemic chemotherapy or transarterial chemotherapy (TACE). Therefore, in this study, we systematically reviewed the existing literature surrounding treatment of unresectable ICCs with yttrium-90 microspheres.

The primary outcomes of this review are survival and radiological response after treatment with local radiation. The overall median survival of patients with unresectable intrahepatic cholangiocarcinoma is 15.5 months from the initiation of yttrium-90 microsphere therapy. Since there were no randomized comparative trials in this review, it is difficult to compare survival with yttrium-90 microspheres to survival after other treatments. However, recently published data show the overall survival of unresectable ICC after systemic cisplatin-gemcitabine chemotherapy is 11.7 months^8^ and survival after treatment with TACE is 13.4 months.^30^ All of these results are higher than historical survival times of less than eight months.^6,7^ Taken together, the results of radioembolization are promising as an alternative therapy for patients with unresectable ICC. However, randomized controlled trials will be required to determine the optimal treatment or combination treatment modality.

As with all systematic reviews, the quality of the combined results is dependent on the quality of the original articles. For example, the overall survival in our pooled analysis is from a heterogeneous population. Some patients underwent systemic chemotherapy prior to yttrium-90 microsphere treatment and some underwent systemic chemotherapy during treatment, however, we only reported survival since the initiation of the yttrium-90 treatment. Therefore, the overall pooled survival may underestimate the effects of radioembolization if some patients have already undergone previous therapy. One study^29^ highlights this point: the one-year overall survival for patients treated with first-line radioembolization was 84.6% compared to 20.2% for those treated with salvage therapy. This study also has the lowest median survival of all included studies. In contrast, selection and publication bias could overestimate the effects of yttrium-90 microspheres treatment, especially in retrospective cohort studies.

Tumour response using RECIST,^22,25,28^ mRECIST^21^ or other radiological criteria^18,19^ was reported in six included studies. In these studies, at three months, partial response was seen in 28% of patients by imaging criteria and stable disease was seen in 54% of patients. Complete responses were only reported in one study where this response was seen in two of nine patients.^19^ However, it should be noted that this is also the only study that used positron emission tomography imaging and response evaluation criteria in solid tumours (PERCIST) criteria to evaluate tumour response.

A potential benefit of radiotherapy with yttrium-90 microspheres is the conversion of unresectable to resectable disease. Surgical resection of previously inoperable disease occurred in seven patients in three different studies. In the study by Mouli et al.,^25^ five patients of their cohort of 46 underwent surgical resection post-treatment with yttrium-90 microspheres. With a median follow-up of over 2.5 years after surgical resection, all five patients were alive. All patients were treatment naïve prior to treatment with yttrium-90 microspheres. Martinez et al.^24^ reported one patient who underwent surgical resection post-radioembolization and had no evidence of recurrent or residual disease; however, the length of follow-up was not specified. The positive results of surgical resection in these studies are in accordance with surgical resection offering the highest survival benefit for patients with ICC.^6,7^ Although there is the probability of publication bias, there is the ability of treatment with yttrium-90 microspheres to downsize tumours to resectability in a minority of patients. Therefore, a potential indication for radioembolization therapy is in cases of borderline resectable tumours at initial presentation.

The overall morbidity and mortality of patients undergoing radioembolization therapy was reported in eight of the 12 included studies (Table 4). Only one death was reported in all of the included studies.^28^ Serious morbidity requiring intervention or long-term sequelae included complications of ulcers (due to bead migration), pleural effusions and ascites. The majority of morbidity was from fever, abdominal pain and nausea. Overall, the complication profile of radioembolization is similar to that of chemoembolization seen in recent systematic reviews of similar disease process.^30,31^

Radioembolization-induced liver disease (jaundice and ascites appearing 1–2 months after radioembolization in the absence of tumour progression or bile duct occlusion^32^) was not specifically mentioned in any study. However, a number of studies did report cases of ascites and hepatitis post-radioembolization, which may indicate radioembolization-induced liver damage did occur. This disease is especially common in radioembolization therapy patients who have previously undergone systemic chemotherapy (which the majority of patients in this review have). Recently, modified radioembolization protocols have been designed to decrease the risk of liver damage post-radioembolization and it is recommended that systemic chemotherapy be delayed by two months.^32^

There are some limitations to the current study. First, five studies were retrospective cohorts which carry the possibility of selection bias. Second, seven included studies were published in abstract form only. Due to their concise nature, abstract publications provide only limited information regarding treatment, follow-up and outcomes. In addition, abstract publications may have not undergone the same stringent peer-review process that a full manuscript has. A dedicated search for the full manuscript of the included abstracts was performed, however, none were found. Third, there is heterogeneity between included studies and no standardized reporting of results. Differences across studies include the type of yttrium-90 radiation microspheres, radiation doses and the length of time of the cohort study. In addition, and likely due to the relative rarity of unresectable ICC, the population within studies is also dissimilar. This heterogeneity includes both patient factors (presence of metastatic disease and size of tumour) and treatment factors (prior chemotherapy or surgical intervention and concomitant chemotherapy). Last, although the search for studies to include in the pooled analysis was stringent, it is possible that some relevant studies were missed.

The pooled analysis of radioembolization therapy with yttrium-90 microspheres for the treatment of unresectable ICC in this study demonstrates an overall survival of 15.5 months. This is higher than historical survival^6^ and is similar to survival with systemic chemotherapy^8^ and TACE therapy.^30^ There is the potential for tumour resectability post-local radiation therapy and the side effect profile is also similar to that seen with TACE therapy for the same disease.^30^ Therefore, the use of yttrium-90 microspheres should be considered in the list of available treatment options for ICC. However, future randomized trials comparing systemic chemotherapy, TACE and local radiation will be required to identify the optimal treatment modality for unresectable cholangiocarcinoma. In addition, the creation of a treatment registry with standardized criteria has been recommended by the Brachytherapy Oncology Consortium.^33^ Report standardization may also allow any synergistic effects from the concomitant use of chemotherapy and Yittrim-90 radiation microspheres to be identified.

## Disclosures

The authors of this manuscript have no conflicts of interest to disclose and there has been no financial support for this research study. DPA, RG and SA participated in study design, performance of research, data analysis and writing the paper. XS and NK participated in performance of research and data analysis. SL participated in study conception, design, data analysis and writing the paper. All authors contributed to critical review of the final manuscript.

## Conflict of interest

The authors of this manuscript have no conflicts of interest to disclose and there has been no financial support for this research study. DPA, RG and SA participated in study design, performance of research, data analysis and writing the paper. XS and NK participated in performance of research and data analysis. SL participated in study conception, design, data analysis and writing the paper. All authors contributed to critical review of the final manuscript.

## Figures and Tables

**Table 1:** Study design and baseline characteristics within included studies for systematic review.

Reference	Publication year	Country	Study interval	Publication type	Study design	Number of patients	Age (years)^a^	Gender (% male)	Diagnosis	Liver involved
Bower and Little	2013	Australia	2002–2012	Abstract	Retrospective cohort	23	62.5	48	ICC	10–70%
Camacho et al.	2013	USA	NA	Abstract	Prospective cohort	21	NA	NA	ICC	NA
Camacho et al.	2013	USA	NA	Abstract	Prospective cohort	9	58	56	ICC	NA
Chaiteerakij et al.	2011	USA	2000–2009	Abstract	Retrospective cohort	20	NA	NA	ICC	NA
Hoffmann et al.	2012	Germany	2007–2010	Full manuscript	Prospective cohort	33	65.2	54.5	ICC + mets	<50%
Hyder et al.	2013	USA	1992–2012	Full manuscript	Retrospective cohort	46	NA	48	ICC + mets	Tumour >5 cm
Martinez et al.	2013	NA	2012	Abstract	Prospective cohort	2	NA	NA	ICC	NA
Mouli et al.	2013	USA	2003–2011	Full manuscript	Prospective cohort	46	68	54	ICC + mets	<25%
Prajapati et al.	2012	USA	2002–2012	Abstract	Prospective cohort	24	NA	NA	ICC	NA
Saxena et al.	2010	Australia	2004–2009	Full manuscript	Prospective cohort	25	57	52	ICC + mets	<50%
Shridhar et al.	2012	USA	2009–2011	Abstract	Retrospective cohort	40	NA	NA	ICC + mets	NA
Turkmen et al.	2013	Turkey	2008–2012	Full manuscript	Retrospective cohort	9	NA	NA	ICC + mets	<70%

NA. not available; ICC. intrahepatic cholangiocarcinoma; mets. metastatic disease present.

a Mean.

**Table 2:** Treatment characteristics within included studies.

Reference	90-Yttrium microsphere	Treatments per patient	Dose (GBq)	Other cancer therapy
Bower and Little	NA	1.04	0.7–2.19^a^	Chemotherapy at discression of oncologist
Camacho et al.	Resin	NA	NA	All patients post-chemotherapy (refractory)
Camacho et al.	Resin	NA	NA	All patients post-chemotherapy (refractory)
Chaiteerakij et al.	NA	1.55	NA	NA
Hoffmann et al.	Resin	1.03	1.54^b^	78.8% post-chemotherapy, 36.6% post-surgery
Hyder et al.	NA	NA	NA	27.8% post-chemotherapy. 11.6% post-surgery
Martinez et al.	Resin	NA	NA	NA
Mouli et al.	Glass	2	3.9 Gy^b^^,^^c^	35% post-chemotherapy, 11% post-surgery
Prajapati et al.	Resin	NA	1.68^b^	All patients post- chemotherapy (refractory)
Saxena et al.	Resin	NA	1.76^b^	72% post-chemotherapy, 40% post-surgery
Shridhar et al.	Glass	NA	NA	48% post-chemotherapy, 8% post-surgery
Turkmen et al.	Glass and Resin	NA	NA	All patients post-chemotherapy (refractory)

GBq, gigabecquerel; NA, not available; Gy, Grey.

a Range.

b Mean.

c Radiation dose reported in Gy, therefore, was not included in weighted analysis.

**Table 3:** Radiological response and survival following treatment with 90-Yttrium microspheres.

Reference	Radiology criteria	Response at 3 months (%)				Follow-up^a^ (months)	Survival^a^ (months)	Comments
		Complete	Partial	Stable	Progress			
Bower and Little	NA	NA	34.7	21.7	NA	NA	7	
Camacho et al.	mRECIST	NA	NA	NA	NA	NA	16.3 (7.2–25.4)	
Camacho et al.	PERCIST	22.2	33.3	33.3	11.1	NA	21.7	
Chaiteerakij et al.	mRECIST	0	100^b^		0	NA	14.6 ± 3.9	
Hoffmann et al.	RECIST	0	36.4	51.5	15.2	10	22	
Hyder et al.	mRECIST	3.1^c^	22.4^c^	61.5^c^	13^c^	NA	11.3	
Martinez et al.	RECIST	NA^c^	NA^c^	NA^c^	NA^c^	NA	NA	1 downstaged to surgery
Mouli et al.	WHO	0	25	73	2	29	5.3 (*n* = 10) 14.4 (*n* = 36)	5 downstaged to surgery
Prajapati et al.	RECIST	NA	NA	NA	NA	NA	11.5	
Saxena et al.	RECIST	NA	24	48	20	8.1	9.3	1 downstaged to surgery
Shridhar et al.	NA	NA	NA	NA	NA	6	7.4	
Turkmen et al.	NA	NA^c^	NA^c^	NA^c^	NA^c^	NA	mean 17.7 ± 3.2	

NA, not available; (m)RECIST, (modified) response evaluation criteria in solid tumours; PERCIST, PET response evaluation criteria in solid tumours; WHO, World Health Organization.

a Median and range or SE, except where indicted.

b Partial and stable responses were pooled.

c Radiological response grouped with other intra-arterial treatment modalities.

**Table 4:** Complications following treatment with 90-Yttrium microspheres.

Reference	Morbidity (%)						Enzyme increases (%)			Other complications/Comments
	Mortality	Fatigue	Abdominal pain	Fever	Nausea	Jaundice	Bili	AST	Alk Phos	
Bower and Little	0	0	0	0	0	0	NA	NA	NA	
Camacho et al.	NA	NA	NA	NA	NA	NA	NA	NA	NA	
Camacho et al.	NA	NA	NA	NA	NA	NA	NA	NA	NA	
Chaiteerakij et al.	NA	39	13	0	NA	NA	35	NA	93	
Hoffmann et al.	0	NA	84.8	NA	60.6	NA	69.7	54.5	NA	
Hyder et al.	NA	17	12	3	6	2	NA	NA	NA	Complications grouped with other modes of intra-arterial therapies
Martinez et al.	NA	NA	NA	NA	NA	NA	NA	NA	NA	
Mouli et al.	NA	54	28	NA	22	NA	7	NA	NA	1 Gastroduodenal ulcer, 2 pleural effusions, 7 ascites
Prajapati et al.	NA	17	17	NA	NA	NA	8.3	NA	NA	1 Duodenal ulcer
Saxena et al.	1	64	40	NA	16	NA	8	0	4	1 Duodenal ulcer, 1 Pulmonary Embolism, 4 ascites, 2 pleural effusion
Shridhar et al.	NA	15	8	NA	NA	5	NA	NA	NA	2 Acute radiation hepatitis, 1 chronic radiation hepatitis
Turkmen et al.	NA	NA	NA	NA	NA	NA	NA	NA	NA	

NA, not available; bili, billirubin; AST, aspartate transaminase: Alk Phos, alkaline phosphatase.
